# Age and gender difference in non-drafting ultra-endurance cycling performance - the ‘Swiss Cycling Marathon’

**DOI:** 10.1186/2046-7648-2-18

**Published:** 2013-06-04

**Authors:** Matthias Zingg, Beat Knechtle, Christoph A Rüst, Thomas Rosemann, Romuald Lepers

**Affiliations:** 1 Institute of General Practice and for Health Services Research, University of Zurich, Zurich, 8091, Switzerland; 2 Gesundheitszentrum St. Gallen, Vadianstrasse 26, St. Gallen, 9001, Switzerland; 3 INSERM U1093, Faculty of Sport Sciences, University of Burgundy, Dijon, 21078, France

**Keywords:** Women, Men, Athlete, Sport

## Abstract

**Background:**

In recent years, there was an increased interest in investigating the gender difference in performance and the age of peak performance in ultra-endurance performances such as ultra-triathlon, ultra-running, and ultra-swimming, but not in ultra-cycling. The aim of the present study was to analyze the gender difference in ultra-cycling performance and the age of peak ultra-cycling performance in the 720-km ‘Swiss Cycling Marathon’, the largest European qualifier for the ‘Race Across America’.

**Methods:**

Changes in the cycling speed and age of 985 finishers including 38 women and 947 men competing in the Swiss Cycling Marathon from 2001 to 2012 covering a distance of 720 km with a change of altitude of 4,993 m were analyzed using linear regression.

**Results:**

The gender difference in performance was 13.6% for the fastest cyclists ever, 13.9% ± 0.5% for the three fastest cyclists ever and 19.1% ± 3.7% for the ten fastest cyclists ever. The gender difference in performance for the annual top three women and men decreased from 35.0% ± 9.5% in 2001 to 20.4% ± 7.7% in 2012 (*r*^2^ = 0.72, *p* = 0.01). The annual top three women improved cycling speed from 20.3 ± 3.1 km h^−1^ in 2003 to 24.8 ± 2.4 km h^−1^ in 2012 (*r*^2^ = 0.79, *p* < 0.01). The cycling speed of the annual top three men remained unchanged at 30.2 ± 0.6 km h^−1^ (*p* > 0.05). The age of peak performance for the ten fastest finishers ever was 35.9 ± 9.6 years for men and 38.7 ± 7.8 years for women, respectively (*p* = 0.47).

**Conclusions:**

The gender difference in ultra-cycling performance decreased over the 2001 to 2012 period in the 720-km Swiss Cycling Marathon for the annual top three cyclists and reached approximately 14%. Both women and men achieved peak performance at the age of approximately 36 to 39 years. Women might close the gender gap in ultra-endurance cycling in longer cycling distances. Future studies need to investigate the gender difference in performance in the Race Across America, the longest nonstop and non-drafting ultra-cycling race in the world.

## Background

In recent years, there was an increased interest in investigating the gender difference in ultra-endurance performances, defined as an endurance performance of 6 h and longer [1]. Ultra-endurance performances were mainly held in swimming [2], cycling [3], running [4], and triathlon [5]. There are differences reported for the gender difference in ultra-endurance performance regarding the length of a race [5-8] and the kind of the discipline [6]. For example, Coast et al. [7] compared the world’s best running performances at distances from 100 m to 200 km. Running speeds were different between the genders, with the average difference being approximately 12.4% faster for men with a significant slope to the speed difference across distances where longer distances were associated with greater gender differences. Knechtle et al. [8] showed that the gender difference in the world’s best performances in ultra-triathlon races was approximately 19% in Double Iron ultra-triathlon covering 7.6-km swimming, 360-km cycling, and 84.4-km running and approximately 30% in Deca Iron ultra-triathlon covering 38-km swimming, 1,800-km cycling, and 422-km running. With increasing length of an ultra-triathlon distance, the world’s best women became slower compared to the world’s fastest men [8]. These results may, however, be confounded by the low number of female finishers in ultra-triathlon races [8].

Regarding single endurance disciplines such as ultra-endurance swimming, female and male performances seemed to be close in both indoor pool swimming [9] and open-water ultra-swimming [2]. In the ‘English Channel Swim’, the annual best performance was not different between the genders [2] but differed in the annual top three swimmers [10]. Additionally, in a 12-h indoor pool swimming, the annual best performance was not different between women and men [9]. For shorter open-water distances, however, the gender difference was unchanged at approximately 11.5% in the 26.4-km open-water ultra-swim ‘Marathon Swim in Lake Zurich,’ Switzerland, held between 1987 and 2011 [11].

For cycling, little is known regarding the gender difference in performance [12]. Schumacher et al. [12] studied the results of the ‘Track Cycling World Championships’ from 1979 to 1999 in 200- and 1,000-m individual and team pursuit races for elite and junior athletes and reported a gender difference of approximately 11% ± 1.8% for all disciplines and at all ages. For ultra-endurance cycling, the gender difference in performance has only been investigated in the cycling split in long-distance triathlons such as the ‘Ironman Hawaii’ [5,6] and in long-distance duathlons such as the ‘Powerman Zofingen’ [13]. In the 180-km cycling split in the Ironman Hawaii, the gender difference was between 12.7% ± 2.0% [5] and 15.4% ± 0.7% [6]. In the 150-km cycling split in the Powerman Zofingen, the gender difference in performance amounted to 17% ± 3% [13]. In running, finish times of women improved relative to men through the 1980s in 100-mile (161-km) ultramarathons in North America, but were then stable over the past two decades with the fastest women running about 20% slower than the fastest men [14]. Similar results were reported for the 78-km ‘Swiss Alpine Marathon’ where the gender difference in performance decreased from 22% in 1998 to 17% in 2011 [4].

Apart from the gender difference in performance, the age of peak endurance performance was of interest for athletes to plan a career. A few studies investigated the age of peak performance in endurance sports [15,16]. For marathoners, the age of peak running performance was reported to be at approximately 30 years for both women and men [15]. In ultramarathoners, the age of peak running performance increased across the years. Eichenberger et al. [4] reported, for the 78-km mountain ultramarathon Swiss Alpine Marathon held in Davos, Switzerland, an increase in the age of the top ten runners from approximately 33 to approximately 37 years over the time span of 14 years.

The gender difference in performance and the age of peak performance were not examined in ultra-cycling performance. Therefore, the aims of the present study were to analyze in the 720-km ‘Swiss Cycling Marathon’ race (a) the gender difference in ultra-cycling performance and (b) the age of peak ultra-cycling performance. The Swiss Cycling Marathon is the largest ultra-cycling marathon held in Europe and the largest European qualifier for the ‘Race Across America’ [17], the longest ultra-cycling race in the world covering approximately 3,000 miles (approximately 4,800 km) [18]. Since the rankings in the Race Across America record only sex and race times but not the age of the finishers, we chose the Swiss Cycling Marathon race to investigate the age of peak performance in ultra-endurance cycling. Regarding present literature suggesting that the gender difference in performance grew with increasing length of an ultra-endurance distance [5], we hypothesized that the gender difference in ultra-cycling performance would be higher than the existing reports on cycling split performances in triathlon [5,6] and duathlon [13]. Considering the age of peak endurance performance, we hypothesized that both women and men would reach their best ultra-cycling performance at the age of approximately 35 years.

## Methods

The data set for this study was obtained from the official home page of the Swiss Cycling Marathon [19] and from the race director. All athletes who ever participated in the Swiss Cycling Marathon between 2001 and 2012 were analyzed regarding participation, performance, and age. The present study was approved by the Institutional Review Board of St. Gallen, Switzerland, with a waiver of the requirement for informed consent given that the study involved the analysis of publicly available data.

### The race

**Figure 1:**
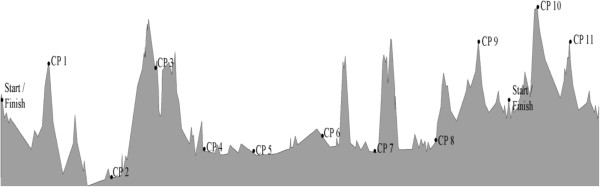
Profile of the course.

The Swiss Cycling Marathon is the largest ultra-cycling marathon in Europe and the largest European qualifier for the Race Across America [19]. The race takes place at the end of June/start of July. For the first loop of 600 km with a total altitude of approximately 4,700 m (Figure 1), the athletes started outside of Berne, Switzerland and cycled to the southern border of Germany. Then they continued along ‘Lake Constance’ towards the Alps of Eastern Switzerland and turned back to Berne. In total, they had to pass 12 checkpoints (Table 1). After the 600-km loop, they had to add an additional loop of 120 km. In 2010, the 600-km loop was reduced to three laps of 200 km due to traffic problems in that year.

**Table 1:** Checkpoints, distance, altitude, and increase and decrease along the track

**Checkpoint**	**Distance (km)**	**Altitude (meters above sea level)**	**Increase (m)**	**Decrease (m)**
Start/finish Ittigen	0	591	0	0
CP 1 Langenbruck	56.7	706	382	254
CP 2 Koblenz	131.6	319	704	979
CP 3 Ewattingen	184.3	714	1,423	1,308
CP 4 Ramsen	242.1	418	1,923	2,102
CP 5 Frasnacht	301.3	409	2,020	2,207
CP 6 Sargans	382.3	485	2,180	2,304
CP 7 Pfäffikon	445.1	414	2,616	2,799
CP 8 Emmenbrücke	518.1	443	3,359	3,506
CP 9 Affoltern	569.1	801	3,922	3,716
Start/Finish Ittigen	605.2	591	4,089	4,089
CP 11 Jassbach	639.1	920	4,610	4,280
CP 9 Affoltern	677.3	801	4,834	4,624
Start/Finish Ittigen	713.4	591	4,993	4,993

*CP* checkpoint.

### Data analysis

In order to increase the comparability of this study with the results from similar studies with data from different races, race times were converted to cycling speed (km h^−1^) by calculating (race distance in kilometer) / (race time in hours). To determine the change in cycling speed over time, the annual top (fastest cyclist for men and women) and the annual top three men and women were analyzed separately. Additionally, the age of the annual top and the annual top three women and men was determined. The gender difference in both cycling speed and age of peak performance was calculated using the equation [(cycling speed or age in women) − (cycling speed or age in men)] / (cycling speed or age in men) × 100. The fastest women and men ever (the course record holder in the regarded time period), the three fastest women and men ever, and the ten fastest women and men ever were determined and compared in order to find the absolute fastest performance and the gender difference of the top finishers.

### Statistical analysis

In order to increase the reliability of the data analyses, each set of data was tested for normal distribution as well as for homogeneity of variances prior to statistical analyses. Normal distribution was tested using D’Agostino and Pearson omnibus normality test, and homogeneity of variances was tested using Levene’s test. To find changes in the development of a variable over time, linear regression was used. To find the differences between two groups a Student’s *t* test was used, with Welch’s correction in case of significant different variances between the two compared groups. Statistical analyses were performed using IBM SPSS Statistics (version 19, IBM SPSS, Chicago, IL, USA) and GraphPad Prism (version 5, GraphPad Software, La Jolla, CA, USA). Significance was accepted at *p* < 0.05 (two-sided for *t* tests). Data in the text are given as mean ± standard deviation (SD).

## Results

### Participation trends

**Figure 2:**
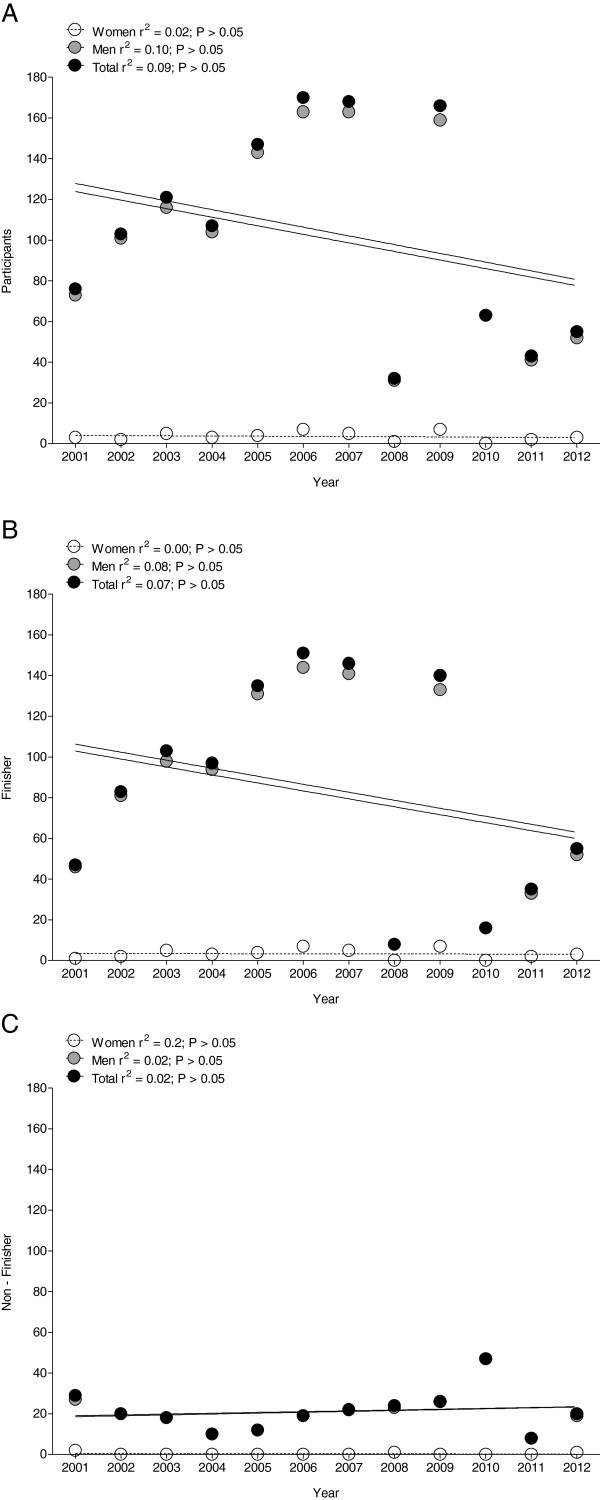
Number of annual female, male, and overall participants (A), annual finishers (B), and annual non-finishers (C).

Between 2001 and 2012, a total of 1,251 athletes started with 1,209 men (96.6%) and 42 women (3.4%). The 1,016 (84%) athletes finished successfully within the time limit. Of the finishers, 977 (96.2%) were men and 39 (3.8%) were women. Across the years, there was no change in the annual number of participants (Figure 2A), the annual number of finishers (Figure 2B) and the annual number of non-finishers (Figure 2C) (*p* > 0.05). The mean annual number of starters was 104 ± 50 with 101 ± 48 men and 4 ± 2 women. The mean annual number of finishers was 85 ± 50 with 81 ± 50 men and 3 ± 2 women. For non-finishers, 21 ± 10 starters were not able to finish, with 21 ± 10 men and 0 ± 1 women. For the starters, 96.6% were men and 3.4% were women. Considering the finishers, 96.2% were men and 3.8% were women and for non-finishers, 98.7% were men and 1.3% were women. Over the years, one woman was able to finish this race two times in 2005 and 2006. All other women finished the race only once.

### Cycling performance

**Figure 3:**
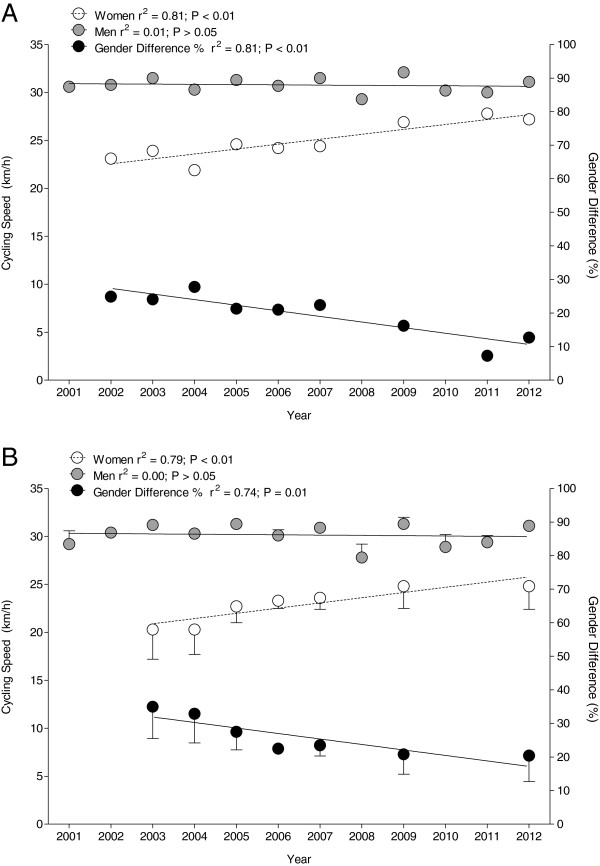
Cycling speeds of the annual fastest women and men (A) and the annual three fastest women and men (B).

For the annual fastest athletes, women have enhanced cycling speed from 23.1 km h^−1^ in 2002 to 27.2 km h^−1^ in 2012 (Figure 3A). The annual fastest men showed no change in cycling speed across years with an unchanged cycling speed of 30.6 ± 0.8 km h^−1^ (*p* > 0.05). The gender difference in performance decreased from 24.9% in 2002 to 12.7% in 2012. For the annual top three finishers, women have improved cycling speed by 22.2% from 20.3 ± 3.1 km h^−1^ in 2003 to 24.8 ± 2.4 km h^−1^ in 2012 (*r*^2^ = 0.79, *p* < 0.01) (Figure 3B). In men, however, cycling speed of the annual top three finishers remained unchanged at 30.2 ± 0.6 km h^−1^ (*r*^2^ = 0.01, *p* > 0.05). The gender difference in performance decreased from 35.0% ± 9.5% in 2003 to 20.4% ± 7.7% in 2012 (−41.7%) (*r*^2^ = 0.74, *p* = 0.01).

**Figure 4:**
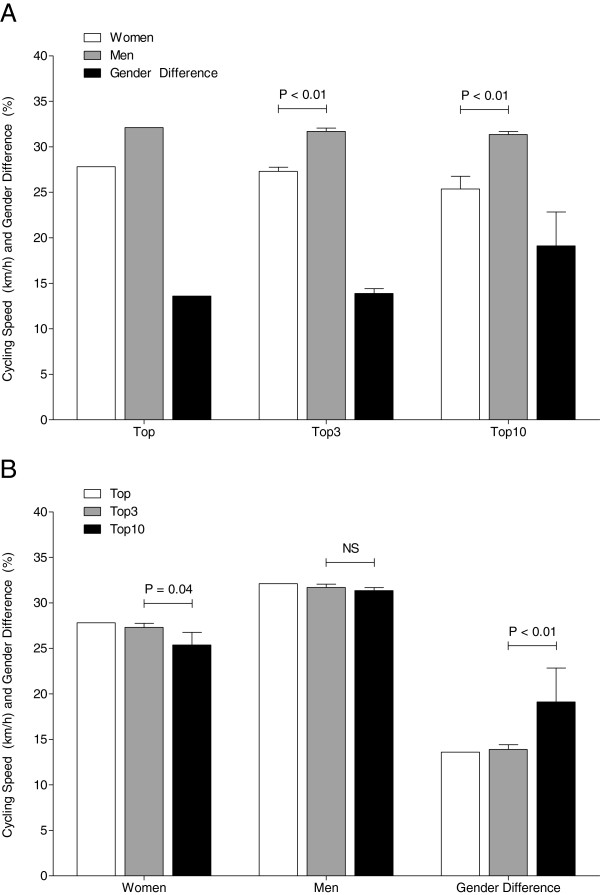
**Cycling speed and gender difference of the fastest ten women and men ever.** Cycling speed and gender difference of the fastest ever, the fastest three ever, and the fastest ten women and men ever, comparing the performance between women and men (**A**) and between the ever fastest three and the ever fastest ten athletes (**B**). *p* Values indicate whether the groups showed a significant difference; *NS* not significant.

When the fastest finishers ever (the course record holder in the regarded time period), the three fastest finishers ever, and the ten fastest finishers ever were compared, the fastest woman ever achieved a mean speed of 27.8 km h^−1^ and the fastest man ever achieved a mean speed of 32.1 km h^−1^ with a gender difference in performance of 13.6% (Figure 4A). For the fastest three women ever, cycling speed was 27.3 ± 0.5 km h^−1^ and for fastest three men ever, at 31.7 ± 0.3 km h^−1^ with a gender difference in performance of 13.9% ± 0.5%. The fastest ten women ever achieved a cycling speed of 25.4 ± 1.4 km h^−1^, and the fastest ten men ever achieved a cycling speed of 31.4 ± 0.3 km h^−1^ with a gender difference in performance of 19.1% ± 3.7%. The fastest three women ever with 27.3 ± 0.5 km h^−1^ were 7.5% faster compared to the fastest ten women ever with 25.4 ± 1.4 km h^−1^ (Figure 4B). The fastest three men ever with 31.7 ± 0.3 km h^−1^ were not faster compared to the fastest ten men ever with 31.4 ± 0.3 km h^−1^. The gender difference in performance for the fastest three men ever was lower (13.9% ± 0.5%) compared to the gender difference in performance for the fastest ten men ever (19.1% ± 3.7%).

### The age of peak performance

**Figure 5:**
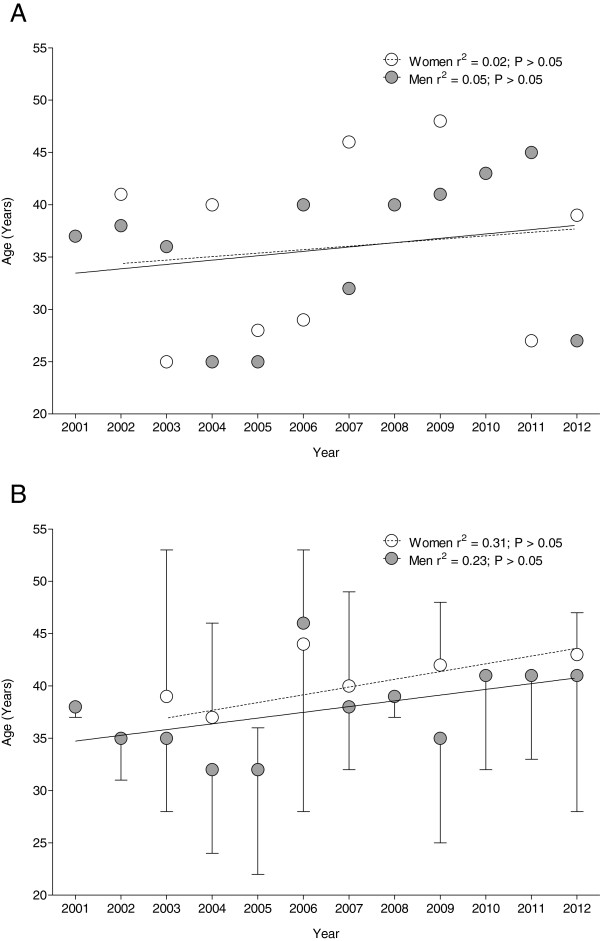
**Difference in age of peak performance of fastest men and women.** Age of the annual fastest women and men (**A**) and the annual three fastest women and men (**B**).

**Figure 6:**
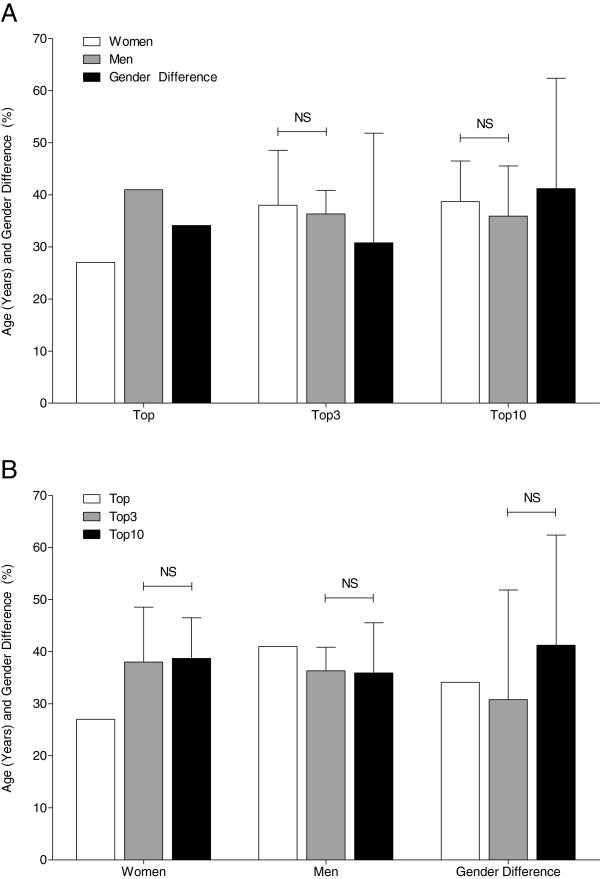
**Age and gender difference of the fastest men and women ever.** Age and gender difference of the fastest ever, the three fastest ever, and the ten fastest ever women and men comparing the age of women and men (**A**) and the age between the fastest three ever and the fastest ten ever finishers (**B**). *p* Values indicate whether the groups showed a significant difference; *NS* not significant.

The age of the annual fastest athletes remained unchanged over time at 33.0 ± 8.7 years for women and 36.0 ± 7.0 years for men (Figure 5A) (*p* > 0.05). Regarding the age of the annual top three cyclists, women and men had the same age of 37.8 ± 9.7 years for women and 37.5 ± 6.8 years for men, respectively (Figure 5B). The fastest woman ever was 27 years old and the fastest man ever had 41 years with a gender difference of 34.1% (Figure 6A). The fastest three women ever were 38 ± 10.5 years old and the fastest three men ever, 36.3 ± 4.5 years, with a gender difference of 30.8% ± 21.0%. Regarding the ten fastest finishers ever, women were 38.7 ± 7.8 years and men 35.9 ± 9.6 years old with a gender difference of 41.2% ± 21.2%. For both three and the ten fastest women and men ever, the age of peak performance was not different between women and men (Figure 6B).

## Discussion

The aims of the present study were to determine (a) the gender difference in ultra-cycling performance and (b) the age of peak ultra-cycling performance in the 720-km Swiss Cycling Marathon, the largest European qualifier for the Race Across America. We hypothesized that (a) the gender difference in ultra-cycling performance would be higher than existing reports on cycling split performances in triathlon and duathlon and (b) the age of peak ultra-cycling performance would be at approximately 35 years. The main findings were that (a) the gender difference in ultra-cycling performance decreased across years for both the annual winners and the annual top three cyclists and (b) the age of peak ultra-cycling performance was at approximately 36 to 38 years and higher than that reported for other endurance disciplines.

### The gender difference in ultra-cycling performance

The comparison of the gender difference in cycling speed showed a difference of 13.6% for the fastest cycling speeds ever, 13.9% ± 0.5% for the three fastest finishers ever, and 19.1% ± 3.7% for the ten fastest finishers ever. A possible explanation for these findings could be the fact of the low female participation in the race and therefore the drop of performance in women was faster [7]. Although these findings in gender difference in performance were in line with the previous results reported for cycling split times in triathlon [5,6] and duathlon [13], only a few studies investigated the change in gender difference in nonstop and non-drafting ultra-cycling performance across years. The higher gender difference in performance in the Swiss Cycling Marathon compared to the cycling split performance in Ironman Hawaii might also be due to the generally lower number of women competing and finishing in ultra-endurance races [8-10,13,14]. Another potential explanation was provided by the findings of Coast et al. [7] showing that the gender difference in performance seemed to increase with the duration of competition in running events between 100 m and 200 km.

The gender difference in endurance performance seemed to be due to biological reasons [20]. For example, in endurance running competitions, the performance depends mainly on both the aerobic capacity and the muscular strength. Since women have a smaller aerobic capacity [21] and a lower muscular strength [22] compared to men, the gap in endurance performances between men and women is unlikely to narrow naturally. Cheuvront et al. [20] reported that the gender difference in running decreased in recent years without the supposed intersection in performance between genders [23]. In several studies investigating runners, cyclists, and swimmers, the gender difference in performance amounted to approximately 11% to 16% [12,15,24]. In city marathon running between 1983 and 2009, men were 11.6% ± 1.8% faster than women [15]. Sparling et al. [25] analyzed the annual world rankings from 1980 to 1996 for 1,500 m and the marathon. In the 1,500 m, the gender difference of 11.1% ± 1.1% in the world’s best times was consistent from 1980 to 1996. In the marathon, the gender difference in world’s best times of 11.2% ± 0.9% was the same as for the 1,500 m distance. In the Track Cycling World Championships from 1979 to 1999, the gender difference in performance was 11% ± 1.8% for all disciplines and all ages [12]. In swimming, the gender difference in performance was 11% ± 1% for 1,500-m freestyle [26]. Eichenberger et al. [4] showed a larger gender difference in performance in 78-km mountain ultramarathoners in the Swiss Alpine Marathon with 16% for winners and 20% for top ten runners. It seemed that the gender difference in performance was growing with increasing duration of the competition but was rather constant for a specific discipline.

The increase in gender difference in performance of approximately 10% to 16% with an increased duration of an endurance performance might be explained by anthropometric differences between female and male endurance athletes. The increase in gender difference with increasing length of an ultra-endurance performance such as an ultra-triathlon [8] or an open-water ultra-swim [10] was most probably due to the lower skeletal muscle mass in women compared to men. It has been shown that male ultra-endurance athletes had a higher skeletal muscle mass than female ultra-endurance athletes [27-29]. For example, male Ironman triathletes with approximately 41 kg of skeletal muscle mass had an approximately 46% higher skeletal muscle mass compared to female Ironman triathletes with approximately 28 kg of skeletal muscle mass [27,28]. For ultra-runners, male ultra-runners with approximately 38 kg of skeletal muscle mass [30] had an approximately 38% higher muscle mass compared to female ultra-runners with approximately 27.4 kg of muscle mass [31]. Another major predictor variable for a successful outcome in endurance competitions is the percentage of body fat [32]. Several studies investigated the correlation between the percentage of body fat and the performance of ultra-endurance athletes [33,34]. Knechtle et al. [35,36] reported the average body fat percentage for female 100-km ultramarathoners at approximately 26.8% (20.0% to 31.4%) or approximately 17.0 kg and for men at approximately 16.1% equal to approximately 11.9 kg. The higher skeletal muscle mass and the lower body fat percentage in men may support the theory of a biological origin of the performance difference in women and men.

### The age of peak ultra-cycling performance

Another major finding was that the age of peak ultra-cycling performance showed no difference between women and men for the three and the ten fastest women and men ever. For both the annual winners and the annual top three finishers, the age of peak ultra-cycling performance remained unchanged across years for both women and men. In both genders, the age of peak performance was at approximately 36 to 38 years, higher than reported for other endurance disciplines.

In other endurance sports, the age of peak endurance performance was reported to be specific for a discipline [15,16]. In marathoners, the age of peak performance was at approximately 30 years for both women and men [15]. Hunter et al. [15] analyzed in seven major marathon events the five fastest men and women (i.e., peak of running speed). Over the time span of almost 30 years, peak marathon running speeds were accomplished at the age of approximately 30 years consistent with the findings of Schulz and Curnow [16] which reported that the age of peak performance in other sports disciplines remained unchanged over years. In ultra-marathon running, however, there seemed to be an increase in the age of peak endurance performance across years. Eichenberger et al. [4] found at the Swiss Alpine Marathon in Davos, Switzerland, an increase in the age of the top ten runners from approximately 33 years to approximately 37 years over the time span of 14 years.

Generally, the age of peak endurance performance was specific to the discipline but largely depending on the duration of a competition. The finding that the age of peak ultra-cycling performance occurred at the age of approximately 38 years is comparable to findings in mountain ultramarathon running [4]. It seemed that the age of peak endurance performance was not different between women and men in most sports but increased with the duration or length of the event. Experience seemed to play a major role in succeeding in ultra-endurance events [17,37] and could therefore be a crucial factor in a 720-km ultra-cycling race such as the Swiss Cycling Marathon. Shaw and Ostrow [38] investigated motivational factors in older, i.e. >35 years, athletes. They reported that motivation was a critical factor in accomplishing ultra-endurance performances. Hodge et al. [39] explored different social factor in athletes aged between 28 and 77 years. They reported for athletes older than 35 years that intrinsic motivation was very high to compete for 20 h without public attention.

### Limitations

This cross-sectional data analysis is limited since aspects such as anthropometry [40,41], physiology [42], training [17,40,41], previous experience [17,43], pacing strategy [42,44], nutrition [3,18,45], fluid intake [46,47], and support during the race [17,40,41] were not included. Another weak point is the fact that the analysis is based on publicly available data, without any explanatory variables. The low number of female participants could have influenced the outcome of the data analysis [48].

## Conclusions

The gender difference in performance increased from the fastest cyclists ever with 13.6% to the ten fastest cyclists ever with 19.1% ± 3.7%. Across years, the gender difference in performance in the top ten athletes decreased from 24.9% (2002) to 12.7% (2012). The age of peak ultra-cycling performance was not different between women and men for both the top three and the top ten female and male finishers. Both genders seemed to peak at approximately 38 years of age in ultra-cycling performance. Men were faster than women in the last 12 years in the Swiss Cycling Marathon. It seems unlikely that women will overtop men in the near future in a nonstop and non-drafting ultra-cycling race of approximately 720 km in length such as the Swiss Cycling Marathon. Women might, however, close the gender gap in ultra-endurance cycling in longer ultra-cycling distances. Future studies need to investigate the gender difference in performance in the Race Across America, the longest ultra-cycling race in the world.

## Competing interests

The authors declare that they have no competing interests.

## Authors’ contributions

All authors designed the study. The manuscript was written by all authors. All authors read and approved the final manuscript.
